# Case Report: Case report: Non-invasive mechanical ventilation in combination with bronchoscopy in the treatment of respiratory failure of lung cancer patient.

**DOI:** 10.12688/f1000research.124457.1

**Published:** 2022-10-03

**Authors:** Katarzyna Guziejko, Łukasz Minarowski, Robert Mróz

**Affiliations:** 1 2nd Department of Lung Diseases and Tuberculosis, Medical University of Bialystok, Bialystok, Zurawia 14, 15-540, Poland

**Keywords:** cancer, respiratory failure, non-invasive ventilation, flexible bronchoscopy, palliative care

## Abstract

**Background:** Respiratory failure (RF) is a common medical problem among cancer patients. Particularly active or ex-smokers diagnosed with chronic obstructive pulmonary disease (COPD) or lung cancer may develop severe hypoxemic and hypercapnic respiratory failure. Moreover, pneumonitis as a complication of the currently widely used immunotherapy of various cancers, may cause respiratory disorders requiring ventilation support. Non-invasive ventilation (NIV) is recommended as the first-line treatment for this type of respiratory failure and reduces the need for endotracheal intubation.

**Case presentation:** We present a case report of lung cancer patient, who received NIV in the treatment of RF due to an infectious exacerbation of COPD. In addition, NIV enabled assisted flexible bronchoscopy (NIV-FB) to be performed. During the procedure tumor samples were collected for further molecular diagnosis of lung cancer. Improvement of the patient general condition and quality of life was also achieved.

**Conclusions:** NIV can be used at any stage of oncological management in patients with lung cancer. It can also be implemented during endoscopic procedures of the respiratory system, as well as support in palliative care of patients with lung cancer at the end of life. Further studies should evaluate the use of NIV in conjunction with various oncological treatments and identify the exact contradictions for BF with NIV support in advanced cancer patients with RF.

## Introduction

Respiratory failure (RF) is one of the most common complications associated with cancer. ^1^ Acute RF is a life-threatening condition that requires immediate treatment. The use of non-invasive ventilation (NIV) reduces the need for endotracheal intubation in these cases, also in lung cancer patients. ^2^^,^^3^ Another common recommendation in clinical practice is the use of NIV in exacerbations of chronic obstructive pulmonary disease (COPD), cardiogenic pulmonary edema, acute lung injury, acute respiratory distress syndrome, bacterial or viral pneumonia, and post-extubation failure. ^4^^–^^6^ Immunotherapy currently plays an important role in the treatment of cancer patients, and the range of indications for this treatment is constantly expanding. ^7^ Pneumonitis, as its complication may also cause respiratory disorders that require non-invasive ventilation. ^8^ Moreover, the implementation of NIV during invasive endoscopic procedures among patients with RF is a new indication for this type of ventilation support ^4^^,^^5^^,^^9^ We present a case of a patient with lung cancer who was treated with NIV in the course of an infectious exacerbation of COPD. NIV allowed for RF management, assisted flexible bronchoscopy (NIV-FB) performance obtaining tissue samples for molecular testing, and improving the patient’s general condition and quality of life. Thanks to this, oncological treatment could be started.

## Case description

A 67-year-old female Caucasian patient, retired teacher, severe cigarette smoker (40 pack-years), with advanced lung cancer (adenocarcinoma cT4N3M1c CS IV), was admitted to the 2nd Department of Lung Diseases and Tuberculosis for oncological treatment. The diagnosis of lung cancer was made in the Department of Internal Medicine of the General Hospital using fine-needle biopsy of metastases to the subcutaneous tissue. No tissue samples were available for molecular diagnostics. Severe acute respiratory syndrome coronavirus-2 infection was ruled out by negative reverse transcription polymerase chain reaction (RT-PCR) on nasopharyngeal swabs.

She had a history of chronic obstructive pulmonary disease (Global Initiative for Chronic Obstructive Lung Disease (GOLD) stage 4D), paroxysmal atrial fibrillation on anticoagulant treatment with apixaban (5 mg, twice a day).

At the time of admission, the patient was in poor condition. The Eastern Cooperative Oncology Group Scale of Performance Status (ECOG PS) was rated at 3. She reported progressive dyspnoea, severe pain in the right scapula and spine of 7/8 points on the numerical rating scale (NRS).

Physical examination revealed tachypnoea (respiration rate 30 breaths per minute), peripheral cyanosis, multiple wheezing and rales on auscultation over the lungs. Oxygen saturation ( *S*pO _2_) was 85% with 6 L per minute oxygen therapy with the nasal cannula (fraction of inspired oxygen ( *F*iO _2_): 0.44).

Laboratory tests have shown elevated levels of C-reactive protein, mild thrombocytopenia. In arterial blood gas (ABG) type 2 RF was found (pH: 7.303; carbon dioxide partial pressure (pCO _2_): 67.6 mmHg; oxygen partial pressure (pO _2_): 66.2 mmHg, HCO _3_: 27.9 mmol/L; SatO _2_: 90.5%).

**Figure 1.**
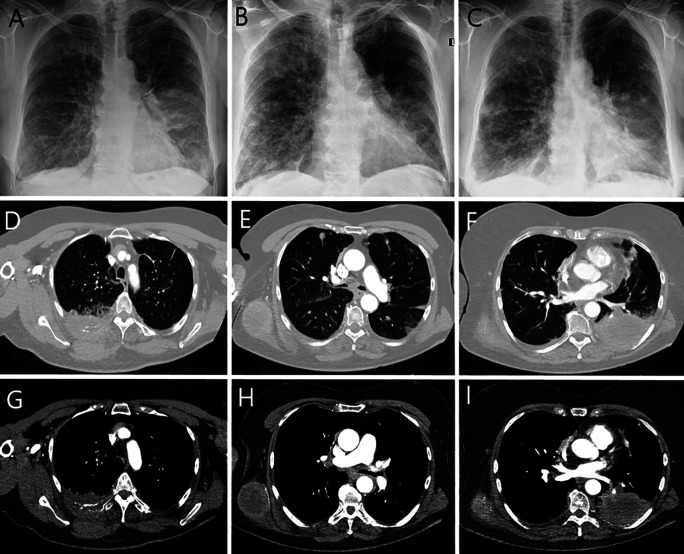
Chest radiograph (A-C): Diffused, patchy opacities and consolidations, bilaterally (more in right lung). Rounded mass in the left lung at the level of the lower pole of the hilum (A). Partial regression of opacities during (B) and after anti-inflammatory treatment (C). **Chest computed tomography (D-F lung window, axial scans; G-I abdomen window, axial scans):** mass of the posterior chest wall on the right side size 7.5×3.4 cm, infiltrating the posterior segment of the fifth rib (D, G); rounded metastasis up to 10 mm in diameter in both lungs, tumor in the right dorsal muscle size 6×5 cm (E, H); tumor in the 6, 9 and 10 segments of the left lung size 9×6×6 cm, infiltrating eighth and ninth rib, with bone destruction (F, I).

Chest radiograph and computed tomography confirmed a tumor in the lower lobe of the left lung, metastases to the right lung, lymph nodes, bones and subcutaneous tissue ( Figure 1).

NIV was implemented for treatment. The target ventilation settings were: spontaneous/timed (S/T) mode, inspiratory positive airway pressure (IPAP) 16 cmH _2_O, expiratory positive airway pressure (EPAP) 7 cmH _2_O, VT 450 mL, *F*iO _2_ 55%, backup rate 14 breaths per minute. For the first three days it was used around the clock, with breaks only for meals, drinking and care activities, during which passive oxygen therapy was used with a nasal cannula ( *F*iO _2_: 0.3). The therapy was well tolerated. ABG control confirmed systematic, gradual improvement (pH: 7.416; pCO _2_: 58.9 mmHg; pO _2_: 66.5 mmHg, HCO _3_^−^: 33.7 mmol/L; SatO _2_: 93.0%). During the next seven days, ventilation time was reduced to the night and approximately 4 hours during the day. Oxygen therapy ( *F*iO _2_: 0.28) was administered for the remainder of the day.

FB at NIV support was also performed during hospitalization. A Philips Respironics Trilogy 100 device was used with a Philips Respironics AF531 naso-oral mask and a bronchoscopy elbow (S/T mode, IPAP 20 cmH _2_O, EPAP 7 cmH _2_O, VT 450 mL, *F*iO _2_: 90–95%, backup rate 18 breaths per minute). The minimum *S*pO _2_ value during the procedure was 85%. A sample was taken by forceps biopsy from a neoplastic mass in the lower lobe of the left lung. Additional molecular testing did not confirm the presence of predictive markers (epidermal growth factor receptor, anaplastic lymphoma kinase, c-ROS oncogene 1) and programmed death receptor-1 expression allowing the use of targeted therapy or immunotherapy.

Pharmacological treatment included broad-spectrum antibiotics (meropenem 3 g intravenously daily, levofloxacin 500 mg twice daily), systemic and inhaled glucocorticosteroids, short acting bronchodilators, mucolytics. Pain management based on NRS was escalated. Combination of non-steroidal anti-inflammatory drugs (ketoprofen 100 mg twice daily), oxycodone with naloxone (20 mg + 10 mg twice daily), fentanyl (50 μg transdermally every 72 hours), pregabalin (150 mg twice daily) and glucocorticosteroid (dexamethasone 8 mg daily) allowed for good pain control (2/3 points on the NRS). Palliative radiotherapy was also performed to the area of the right scapula and surrounding chest wall (a single dose of 8 Gy in one fraction).

**Figure 2.**
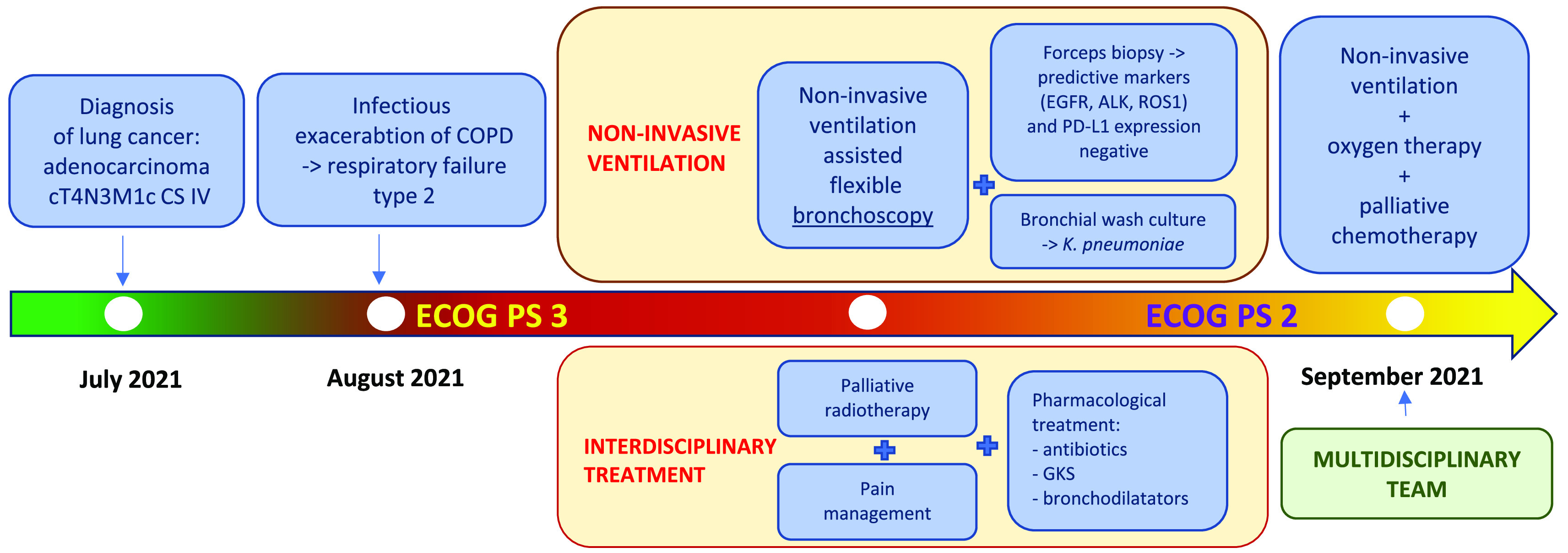
A timeline with all relevant data from this clinical case.

Combined pharmacological treatment, NIV and palliative radiotherapy of the chest wall significantly improved the patient’s condition. ECOG PS 2 has been achieved. After an acute phase of an infectious exacerbation of COPD complicated with type 2 respiratory failure, the patient was qualified to palliative chemotherapy based on the decision of multi-disciplinary team, initially with pemetrexed as monotherapy. NIV and oxygen therapy were continued on an outpatient basis. A timeline with all relevant data from this clinical case is available in Figure 2.

## Discussion

Cancer complications may affect nearly one in four cancer patients. Among them RF is one of the most common. A study of 2028 patients with solid tumors and hematological malignancies showed that complications were more common in patients with metastatic solid tumors, especially lung and breast cancers. Moreover, RF was independently associated with in-hospital mortality. ^1^ Previous studies have shown that NIV is effective in COPD exacerbations, cardiogenic pulmonary edema, acute lung injury, acute respiratory distress syndrome, bacterial or viral pneumonia, and post-extubation failure. ^4^^–^^6^^,^^10^

Unfortunately, NIV-assisted palliative care at the end of life is not a fully researched issue. ^3^ In a randomized feasibility study by Nava *et al*., ^11^ 200 participants with solid tumors and acute respiratory failure and a life expectancy of less than 6 months were randomized to receive either NIV or oxygen treatment. The study found that NIV is more effective than oxygen in reducing dyspnoea and the dose of morphine needed in patients with end-stage cancer. ^11^ A study by Wilson *et al*. ^12^ showed that many patients with do-not-intubate order who received NIV survived until hospital discharge. Similar results have been observed in a Kızılgöz *et al*. ^3^ study. Most lung cancer patients, even those with short life expectancy, who received NIV as supportive therapy, were discharged from the hospital.

In the described case, the NIV treatment of RF in the course of an infectious exacerbation of COPD resulted in a reduction in dyspnoea. Moreover, the patient’s clinical condition improved from 3 to 2 according to ECOG PS. The daily ventilation time was determined on the basis of the ABG values. NIV therapy and oxygen therapy were continued on an outpatient basis.

However, little is known about the use NIV during endoscopic procedures of the respiratory system in patients with RF. ^5^^,^^13^ The use of NIV may enable management of patients who are initially ineligible for invasive respiratory diagnostics. ^14^

In our report, the patient at the beginning of NIV therapy, was unstable in the respiratory system due to high hypercapnia. Effective ventilation allowed to reduce the partial pressure of carbon dioxide and maintain its stable level in ABG control. NIV combined with comprehensive analgesic and anti-inflammatory treatment reduced the severity of symptoms and allowed FB to be performed, which initially seemed impossible. Ventilation settings have been individualized based on clinical data and ABG values. Molecular tests on tissue samples collected during the procedure determined the type of lung cancer treatment.

Ultimately, we do not yet have enough information about the risks and complications of NIV-FB. The narrowing of the tracheal lumen by a bronchoscope may lead to RF exacerbation, which may cause further complications. ^15^ Studies conducted on small groups of patients have shown that NIV-FB is safe, helps to avoid intubation, has a low complication rate, but should be performed by experienced teams. ^16^ However, there are still no precise recommendations regarding NIV-FB in high-risk patients, including RF. ^10^ In a study involving 50 patients, Skoczyński *et al.* also confirmed that NIV-FB is safe even in severely ill patients, but the risk associated with the procedure should always be taken into account. ^5^ They highlight the need of further studies in large study groups to assess the exact level of hypoxemia and hypercapnia, the risk of NIV-FB, setting modalities, indications and contraindications.

Our case emphasizes the key importance of the correct treatment of RF in cancer patients with NIV. Effective NIV therapy allowed not only to reduce dyspnoea, but also to perform FB in a patient previously disqualified due to RF and possible complications. The achieved clinical improvement influenced the further oncological treatment.

## Conclusions

NIV can be the mainstay of first-line treatment of acute or chronic respiratory failure. In addition, it can be used at any stage of oncological management in patients with lung cancer. NIV can also be implemented during endoscopic procedures of the respiratory system, as well as support in palliative care of patients with lung cancer at the end of life. Further research should evaluate the use of NIV in conjunction with various oncological treatments and identify the exact contradictions for FB with NIV support in RF patients with advanced cancer.

## Consent for publication

Written informed consent for the publication of identifying images or other personal or clinical details of participant that compromise anonymity was obtained.

## Data availability

No data are associated with this article.

## Authors’ contributions

KG, ŁM, RMM – analyzed and prepared the data, wrote the manuscript; ŁM – was the leading doctor; KG, RMM – was involved in the clinical management of the patient. Our manuscript reporting adheres to CARE guidelines. All authors read and approved the final manuscript. Katarzyna Guziejko and Łukasz Minarowski contributed equally to the manuscript.
