# Supplementary material for: Case Report: Case report: Non-invasive mechanical ventilation in combination with bronchoscopy in the treatment of respiratory failure of lung cancer patient
Source: F1000Res. 2022 Oct 3;11:1130. [Version 1] doi: 10.12688/f1000research.124457.1 (PMC10439354; doi:10.12688/f1000research.124457.1)
Supplement: Peer review history

# Peer review: Reviewer response for version 1

**DOI:** 10.5256/f1000research.136651.r191677

**Published:** 2023-08-18

**Authors:** Jacek Nasiłowski

**Affiliations:** 1 Department of Internal Medicine, Pulmonary Diseases and Allergy, Medical University of Warsaw, Warsaw, Poland

Guziejko and coworkers presented the exciting case of a COPD patient with stage IV lung cancer who developed hypercapnic respiratory failure due to exacerbation of COPD. During treatment, diagnostic flexible bronchoscopy NIV-assisted was performed to perform molecular investigation looking for other therapeutic options for treating advanced cancer.

 The procedure was uneventful, proving the efficacy of NIV in endoscopic procedures of airways, even in acute exacerbation.

 The minor points of criticism are the following:

1. The authors gave a very rough estimation of the severity of COPD (GOLD 4D), which refers to the former version of GOLD before 2023. I propose adding the most recent result of the patient's spirometry, namely FEV1.
2. I would avoid giving FiO2 when the patient has oxygen therapy by nasal cannula because it only estimates FiO2, not an exact value.
3. What kind of NIV machine was used for the treatment before bronchoscopy? Was it the same, Trilogy 100?
4. In the ST mode of ventilation, Vt is not titrated. The value of Vt changes from breath to breath depending on pressure support, work of breathing and resistance and compliance of the respiratory system.
5. The sequence of the chest X-rays should be given (e.g.day of hospitalization).
6. If chest X-ray A was done on admission, you can note the development of new opacities in the subsequent figures. Was it hospital-acquired pneumonia?
7. If it was the case, the patient did not suffer only from exacerbation of COPD but from pneumonia as well.
8. The description of the chest x-ray and CT scan is not entirely informative. The particular described findings should be enhanced with the markers (arrows, circles) on the pictures.
9. The choice of antibiotics should be explained because the combination of meropenem and levofloxacin is not the first choice of treatment for pneumonia.
10. The information about bronchoscopy is given before information about antibiotics treatment. Did pneumonia develop after bronchoscopy? Could pneumonia be a consequence of the procedure?
11. The NIV-assisted bronchoscopy should be described more precisely: what day of hospitalization, what was the blood gas analysis at the time of bronchoscopy, was there a need to change the ventilation settings after insertion of the bronchoscope, how long was the procedure, was sedation necessary, etc.

Are enough details provided of any physical examination and diagnostic tests, treatment given and outcomes?

Partly

Is the case presented with sufficient detail to be useful for other practitioners?

Partly

Is sufficient discussion included of the importance of the findings and their relevance to future understanding of disease processes, diagnosis or treatment?

Yes

Is the background of the case’s history and progression described in sufficient detail?

Yes

Reviewer Expertise:

NA

I confirm that I have read this submission and believe that I have an appropriate level of expertise to confirm that it is of an acceptable scientific standard, however I have significant reservations, as outlined above.

# Peer review: Reviewer response for version 1

**DOI:** 10.5256/f1000research.136651.r191698

**Published:** 2023-08-04

**Authors:** Habib Md R. Karim

**Affiliations:** 1 All India Institute of Medical Sciences, Raipur, India

I read the manuscript with interest and applaud the work. While NIV use in lung cancer and palliative patient is not unknown, nor is the endoscopy use during NIV, the case bears importance because such literature is still minimal. The authors have used NIV to manage RF arising from AECOPD and have performed bronchoscopy during ongoing NIV.

 The title is, however, not distinct to indicate so, and the title delivers as if both NIV and bronchoscopy were continuous throughout. So, I suggest revising the title.

 Further, the author should indicate the need for sedation during the bronchoscopy, monitoring of NIV tolerance, and success/failure during the therapy.

 In my opinion, the author can also indicate the histopathology report and follow-up of the patient details, if they have any. 

 Best of luck

Are enough details provided of any physical examination and diagnostic tests, treatment given and outcomes?

Partly

Is the case presented with sufficient detail to be useful for other practitioners?

Partly

Is sufficient discussion included of the importance of the findings and their relevance to future understanding of disease processes, diagnosis or treatment?

Yes

Is the background of the case’s history and progression described in sufficient detail?

Yes

Reviewer Expertise:

Noninvasive Ventilation, Pain, Sedation, Perioperative Care, Critical Care

I confirm that I have read this submission and believe that I have an appropriate level of expertise to confirm that it is of an acceptable scientific standard, however I have significant reservations, as outlined above.

# Peer review: Reviewer response for version 1

**DOI:** 10.5256/f1000research.136651.r152867

**Published:** 2022-11-07

**Authors:** Artur Szlubowski

**Affiliations:** 1 Endoscopy Unit, John Paul II Hospital, Kraków, Poland; 2 Endoscopy Unit, John Paul II Hospital, Zakopane, Poland

- A presented case report of a lung cancer patient, who received NIV in the treatment of RF due to an infectious exacerbation of COPD, is well reported. 
- Still, little is known about the use of NIV during endoscopic procedures of the respiratory system in patients with RF.
- Moreover there are still no precise recommendations regarding NIV-FB in high-risk patients.
- It would be nice to add what was the final histopathological result of the neoplastic mass based on forceps biopsy. 

Are enough details provided of any physical examination and diagnostic tests, treatment given and outcomes?

Yes

Is the case presented with sufficient detail to be useful for other practitioners?

Yes

Is sufficient discussion included of the importance of the findings and their relevance to future understanding of disease processes, diagnosis or treatment?

Yes

Is the background of the case’s history and progression described in sufficient detail?

Yes

Reviewer Expertise:

bronchology, including all endosonographic methods

I confirm that I have read this submission and believe that I have an appropriate level of expertise to confirm that it is of an acceptable scientific standard.
